# A Fingerprint Encryption Scheme Based on Irreversible Function and Secure Authentication

**DOI:** 10.1155/2015/673867

**Published:** 2015-03-22

**Authors:** Yijun Yang, Jianping Yu, Peng Zhang, Shulan Wang

**Affiliations:** ATR Key Laboratory of National Defense Technology, Shenzhen University, Shenzhen 518060, China

## Abstract

A fingerprint encryption scheme based on irreversible function has been designed in this paper. Since the fingerprint template includes almost the entire information of users' fingerprints, the personal authentication can be determined only by the fingerprint features. This paper proposes an irreversible transforming function (using the improved SHA1 algorithm) to transform the original minutiae which are extracted from the thinned fingerprint image. Then, Chinese remainder theorem is used to obtain the biokey from the integration of the transformed minutiae and the private key. The result shows that the scheme has better performance on security and efficiency comparing with other irreversible function schemes.

## 1. Introduction

Biometric feature recognition is the technology using different biometric features or personal behaviors from an individual to identify one person. Comparing to other biometric features, fingerprint recognition technology has many advantages. It is common, stable, and precise and cannot be easily faked. The probability of finding two selfsame fingerprints is merely one in five billion. Therefore, it becomes the most widely applied technology in the biometric feature recognition field, and it makes life more convenient and secure. However, since the fingerprint recognition has been applied in many fields, more and more attackers have emerged and the security has been fiercely threatened. Furthermore, fingerprint will stay the same in all lifetime, which means, in case the fingerprint information has been revealed, it is no longer safe permanently. To assure the security of fingerprint recognition, fingerprint encryption technology has been developed.

There are some significant achievements on Fuzzy Vault in the past several years. In 2002, the conception of Fuzzy Vault was proposed by Juels and Sudan [1], who provide an effective private key combining algorithm. Then, the biometric encryption becomes a hot issue in the world. Based on that, Clancy et al. [2] bring up the conception of “fingerprint vault” although the hypothesis of its noise distribution may not be practicable. Uludag and Jain give the definition of Fuzzy Vault for fingerprint [3] and point out that the fingerprint vault in Clancy's authentication algorithm will be affected if the image has been moved or rotated. They firstly use the helper data to verify the fingerprint.

All of these researches have not considered the security of fingerprint template itself. Subsequently, Nandakumar et al. [4] propose a Fuzzy Vault encryption algorithm based on password where the transformed fingerprint vault stores the transformed minutiae, not the original ones. The system is under double protection and the private key will not be revealed until the attackers have breached this double protection at the same time. Caixia and Lin [5] encrypt the minutiae with password. Their encryption algorithm is just to do simple exclusive-or calculation to the coordinate of each minutia, and then joint the transformed minutiae in series. However, the property of irreversibility has not been proved in their paper. Zhang et al. [6] propose a cancelable fingerprint Fuzzy Vault scheme and build an irreversible function based on password. Then, they use the transformed minutiae to compose the fingerprint vault. The principal part of the irreversible function is SHA256 which cannot guarantee the irreversibility. In order to decrease the FRR (false reject rate) and FAR (false accept rate), the time complexity is increased in all above-mentioned Fuzzy Vault scheme based on password.

This paper is organized as follows. Firstly, the minutiae are extracted from the thinned fingerprint image. In order to weaken the influence from noises, a threshold is set to remove the fake minutiae. Secondly, an irreversible function is designed to protect the security of fingerprint. At last, the Chinese remainder theorem (CRT) is used for both the private key binding and the private key recovering. Then, the cancelable fingerprint vault is encrypted in order to guarantee the security of private key and fingerprint vault in storage.

## 2. Preliminary

### 2.1. Fuzzy Vault

**Figure 1:**
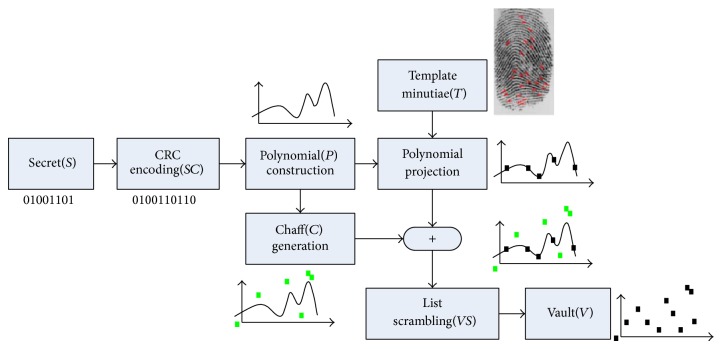
Encryption of Fuzzy Vault for fingerprint.

**Figure 2:**
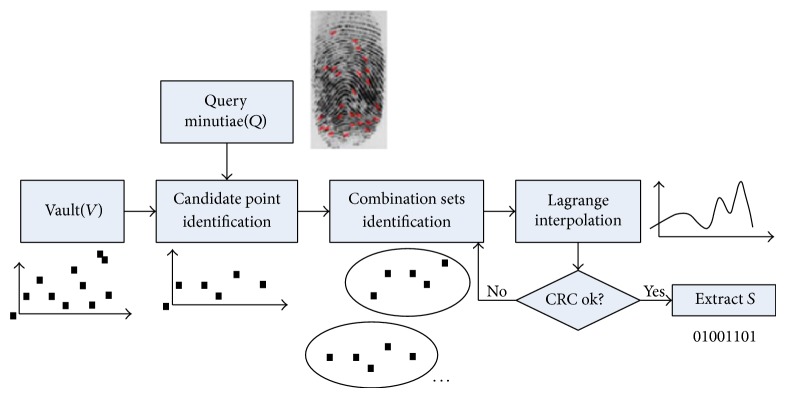
Decryption of Fuzzy Vault for fingerprint.

The Fuzzy Vault algorithm can be divided into two steps. Figures 1 and 2 show the encryption processing and decryption processing of Fuzzy Vault, respectively.

*Encryption*. *S* is the private key. It is encrypted by user 1 with vault *A* (short for fingerprint vault *A*). Firstly, *S* is divided into *n* average parts; each part becomes a coefficient of the polynomial. Use that polynomial to calculate *P*(*A*) and obtain the pair set (*A*, *P*(*A*)). Then, a large amount of random noises are created (the number of noises is 10 times larger than the real minutiae); the real minutiae and noises are mixed up to compose vault *A*.

*Decryption.* In order to obtain the private key that user 1 hides in vault *A*, user 2 has to guarantee that set *B* used to unlock vault *A* has enough superposition with vault *A*. Otherwise, it is quite difficult to rebuild the polynomial *P*. Finally, the RS code is used for decreasing the noise.

### 2.2. The Hash Function

The hash function has been deployed as an important component in information security and cryptography. It takes a message *M* less than 2^64^ bits and produces a hash value with fixed length. It can be defined as follows:

(1) $$\begin{array}{c} H:M⟶h. \end{array}$$

A good hash function should be irreversible and anticollision.

If a hash function is anti-strong collision, it is also irreversible. Assume that *h* is a hash function and Λ is the oracle which can obtain *x* when the input *y* = *h*(*x*) is given. In other words, this oracle can break the property of irreversibility and can obtain another *x*_0_ to the same input where *y* = *h*(*x*_0_). Since the input of hash function is random, a strong collision of *h* is found when *x* ≠ *x*_0_.

There are some common hash functions, such as MD, RIPEMD, and SHA. The SHA1 algorithm has been designed by NIST in 1995. It has been applied widely. The following is the process of SHA1.

**Step 1 .**

The input message should be padded and then processed in 512-bit blocks. Each block is divided into 16 parts of 32-bit length, and finally obtain 80 message words *W*[*j*]  (0 ≤ *j* ≤ 79).

**Step 2 .**

Initialize the five 32-bit registers *A*, *B*, *C*, *D*, and *E* as the temporary memorizer of the 160-bit output.

**Step 3 .**

There are 80 iterations in 4 rounds where each round has 20 iterations. The iteration of the *j*th step in the *i*th round can be described as

(2) Aj+1=AjROTL5+fiBj,Cj,Dj+Ej+Wj+Ki;Bj+1=Aj;Cj+1=BjROTL30;Dj+1=Cj;Ej+1=Dj.

*ROTL* ^*w*^ denotes a left bit rotation by *w* places, *W*[*j*] is the expanded message word of round *j*, and *K*_*i*_ is the round constant of round *i*. *f*_*i*_(*B*_*j*_, *C*_*j*_, *D*_*j*_) is a nonlinear function which is different in 4 rounds.

**Step 4 .**

The 160-bit output of the last block becomes the input of the next block. After processing the last block, the registers *A*, *B*, *C*, *D*, and *E* add their original value, respectively. Then, the 160-bit chaining variable is the final outcome of SHA1.

### 2.3. The Small Integer Solution Problem *SIS*_*p*,*m*,*χ*_

Let *k* be the security parameter. Define an integer *p*, a matrix *F* ∈ *Z*_*p*_^*k*^, and a real number *χ*. The solution of this problem is to find a nonzero integral vector *z* ∈ *Z* ^*m*^(‖*z*‖ ≤ *χ*), which makes *F* × *z* = 0mod⁡*p*.

### 2.4. The $GAPSVP_{14\pi \sqrt{k}\chi }$ Problem

Let *k* be the security parameter. The input of $GAPSVP_{14\pi \sqrt{k}\chi }$ is a *k*-dimension lattice with the basis of *B*. If *λ*(*B*) ≤ *d*, the output is “YES.” If *λ*(*B*) > *γ* × *d*, *γ* > 1, the output is “NO.”

To any random polynomial function *χ*(*k*), *m*(*k*), *p*(*k*) = *k* ^*O*(1)^, if $p\geq 4\sqrt{m}k^{1.5}\chi$ and $\gamma =14\pi \sqrt{k}\chi$, the security of *GAPSVP*_*γ*_ is equivalent to the difficulty of *SIS*_*p*,*m*,*χ*_.

### 2.5. CRT

CRT is one of the famous theorems in mathematics. It can be described as follows. Let *M* = *m*_1_ × *m*_2_ × ⋯×*m*_*k*_. These *k* positive integers *m*_1_, *m*_2_,…, *m*_*k*_ are relatively prime to each other. For any big integer *X*mod⁡*m*_*i*_ = *a*_*i*_  (*i* = 1,2,…, *k*), it can be calculated as *X* = (∑_*i*=1_^*k*^(*M*/*m*_*k*_)*e*_*k*_ *a*_*k*_)mod⁡*M*, where (*M*/*m*_*i*_)*e*_*i*_ = 1mod⁡*m*_*i*_.

## 3. The Proposed Irreversible Fingerprint Encryption Scheme

In order to resist the multiple templates attack, a cancelable Fuzzy Vault scheme based on irreversible function has been proposed in this section. Firstly, the real minutiae are extracted from the registered fingerprint image, and then an irreversible function using different parameters has been designed. An improved SHA1 function has been proposed to transform the original minutiae. In the following section, the positive integers which are relatively prime to each other are created, and the CRT is used to combine the private key with the transformed fingerprint vault and finally the cancelable fingerprint vault is obtained.

### 3.1. The Improved Fingerprint Minutiae Extraction Algorithm

#### 3.1.1. The Traditional Minutiae Extraction Algorithm

The minutiae extraction algorithm can mainly be divided into two different categories: extracting minutiae from the thinned fingerprint image or extracting minutiae from the original fingerprint image directly. Besides, [7] also provides a new image extracting method. The disadvantage of the first category is that it will create lots of fake minutiae and consume a lot of time. The disadvantage of the second category is that it has a poor performance on low-quality fingerprint image.

**Figure 3:**
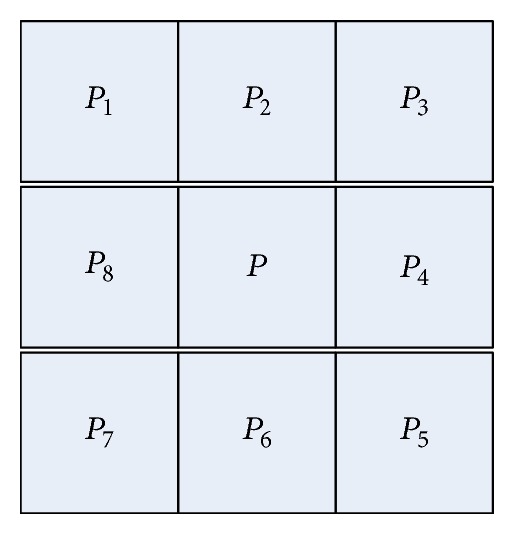
The 8 adjacent pixels of *P*.

Since the low-quality images are very common, this paper extracts minutiae from the thinned fingerprint image. As shown in Figure 3, let *P* be the object pixel, and then there are *P*_1_,…, *P*_8_ surrounding it. These eight points are defined as the eight adjacent pixels of *P*.

Define

(3) TsumP=∑i=18Pi,      TsubP=∑i=18Pi+1−PiP9=P1.

For any random pixel *P*, if *T*_sum_(*P*) = 1 or *T*_sub_(*P*) = 2, *P* is the termination minutia of the ridge line. If *T*_sum_(*P*) = 3 or *T*_sub_(*P*) = 6, *P* is the bifurcation minutia of the ridge line.

#### 3.1.2. The Removal of Fake Minutiae

The minutiae after extraction may contain many fake minutiae. In this paper, the fake minutiae can be removed after the minutiae extraction from the thinned fingerprint image. Defining a threshold *D*, when the distance of any two minutiae is less than *D*, these two minutiae should be removed as follows.

1. If the two minutiae are both termination minutiae and have almost the same orientation, these two fake minutiae are formed from short lines or gap.
2. If the two minutiae are both bifurcation minutiae, these two fake minutiae are formed from holes or conjoint lines.
3. If one minutia is a termination minutia and the other is a bifurcation minutia, these two fake minutiae are formed from burr.

**Figure 4:**
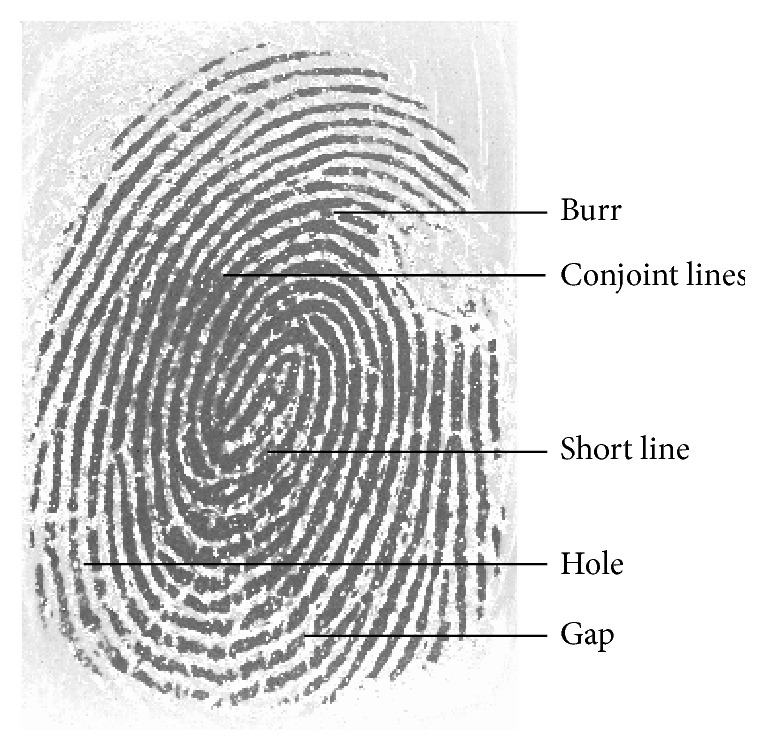
Noises of the thinned fingerprint image.

All the above-mentioned fake minutiae have been shown in Figure 4.

As it is shown in Table 1, although the fake minutiae removal processing will remove a few real minutiae, it can remove almost the entire fake minutiae and thereby effectively decrease the FAR.

The noise can also be characterized by data processing which aims to extract useful information from mass data and eliminate redundancy. Li [8, 9] proposes a class of negatively fractal dimensional Gaussian random functions to eliminate the useless data. The properties of the generalized Cauchy distribution have been analyzed in his earlier paper [10]. Cattani et al. have built a low-complexity separable mathematical model, and then they discuss the efficiency in their paper [11]. The noise in fingerprint can be suppressed once its character is extracted.

### 3.2. The Irreversible Function

#### 3.2.1. The Improved Hash Function

Here is an essential on the collapse to SHA1. As long as step function (4) can be denoted by a formula containing message word *W*[*j*], the differential can always be eliminated gradually by modular differential method. In 2005, Professor Wang et al. [12] successfully found a local collision and consequently obtained the collision to SHA1 with less time complexity than the birthday attack. The local collision in the second round iteration of SHA1 can be shown as follows.

The step function of SHA1 in the second round is

(4) Aj+1=AjROTL5+Bj⊕Cj⊕Dj+Ej+Wj+K2;

(5) Bj+1=Aj;

(6) Cj+1=BjROTL30;

(7) Dj+1=Cj;

(8) Ej+1=Dj.

Suppose there is no differential from the beginning to the (*j* − 1)th step of the second round. Then, in the *j*th step of the second round, a 1-bit differential is brought in. The first bit of the register *A* changes from 0 to 1 (or 1 changes to 0). According to formulae (4)–(8), it is obvious that there is merely differential in register *A*. In registers *B*, *C*, *D*, and *E*, there will only be the evaluation or circularly left-shift calculation, which cannot create any differential. Formula (4) contains the message word *W*[*j*]; therefore, *W*[*j*] can be denoted by

(9) $$\begin{array}{c} W\left[j\right]=A_{j+1}-\left(A_{j}ROTL^{5}\right)-\left(B_{j}⊕C_{j}⊕D_{j}\right)-E_{j}-K_{2}. \end{array}$$

In this formula, the differential in the right side can be inferred from the last chaining variables. The attacker can modify the message word *W*[*j*] to eliminate the differential step by step. According to Table 2, the differential of step *j* can be eliminated gradually in step *j* + 6.

In Table 2, *W*[*j*] indicates the bits which the attacker modifies in *W*[*j*].

The security has been fiercely threatened by above-mentioned local collision and therefore the SHA1 algorithm should be improved to resist this modular differential attack. Consequently, an anticollision SHA1 algorithm has been proposed. In this paper, formula (4) has been changed into

(10) Aj+1=Aj+WjROTL5+fjBj,Cj,Dj +Ej+WjROTL17+Kj.

**Figure 5:**
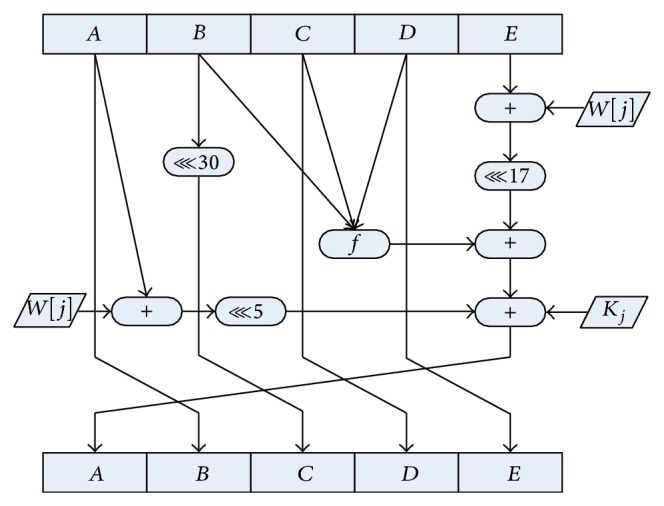
The iteration of improved SHA1.

Formulae (5)~(8) remain unchanged. In formula (10), since there are two message words in *A*_*j*+1_, it becomes very difficult to denote *W*[*j*] by a formula. 17 (the circularly left-shift bit in formula (10)) is relatively prime to 5, 30 (the circularly left-shift bit in formulae (4) and (6)), and 32 (the length of chaining variables). In this condition, it becomes more difficult to eliminate the modular differential by modular differential attack. The iteration of improved SHA1 has been shown in Figure 5.

#### 3.2.2. The Construction of Minutiae Irreversible Transforming Function

Suppose the minutiae set is {*x*_*i*_, *y*_*i*_, *θ*_*i*_}, *i* = 1,2,…, *k*. (*x*_*i*_, *y*_*i*_) and *θ*_*i*_ are the coordinate and orientation of the *i*th real minutia, respectively. The value of minutiae can be set into an appropriate range according to different requirements. In this paper, the value of *x*_*i*_, *y*_*i*_, *θ*_*i*_ is restricted from 0 to 255 (let *q* = 256).

Let *R* be a random number, and let *M* be a $\tilde{m}\times \tilde{n}$ matrix. In this paper, the size of *M* has been defined as 3 × 3. Firstly, calculate *h* = *H*(*R*), where the hash function is the improved SHA1 algorithm described in Section 3.2.1. The length of *h* is 160 bits. Then, create a three-dimensional array (*a*, *b*, *c*). Define the lowest bit of *h* as bit 1, and *a* is a 24-bit variable created from the first 24 bits of *h*. The variables *b* and *c* are created from bits 25–48 and bits 49–72 of *h*, respectively.

Afterwards, execute the following transforming to every minutia {*x*_*i*_, *y*_*i*_, *θ*_*i*_}, *i* = 1,2,…, *k*, as follows:

(11) $$\begin{array}{c} \left(\begin{array}{c} u_{i}\\ v_{i}\\ \varphi_{i} \end{array}\right)=\left[M\left(\begin{array}{c} x_{i}\\ y_{i}\\ \theta_{i} \end{array}\right)+\left(\begin{array}{c} a\\ b\\ c \end{array}\right)\right]\mathrm{mod}q. \end{array}$$

**Figure 6:**
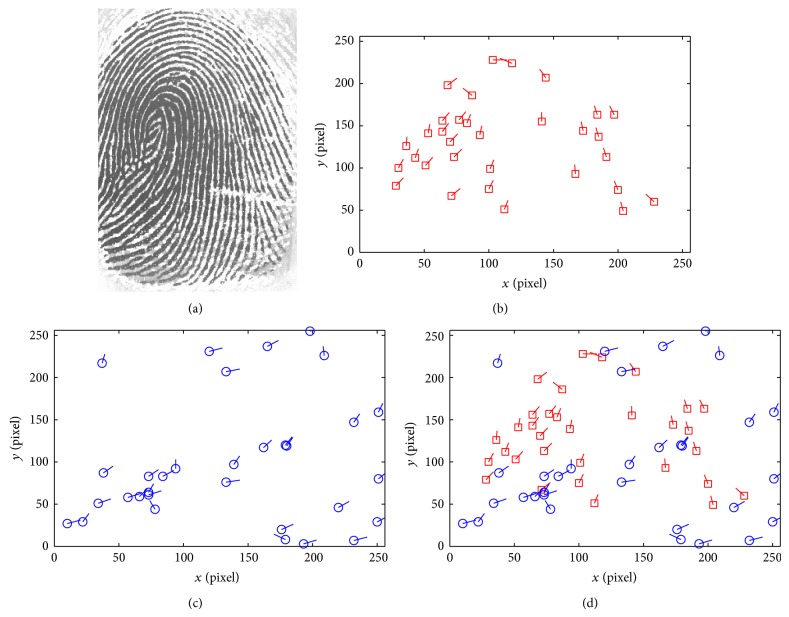
The original fingerprint image and its minutiae. (a) The original fingerprint image. (b) The minutiae of the original minutiae. (c) The minutiae after transforming. (d) The combination of two templates.

The transformed set {*u*_*i*_, *v*_*i*_, *φ*_*i*_}, *i* = 1,2,…, *k*, is treated as the new real minutiae set. Randomly choose *R* = *iscbupt*, $M=\left[\begin{array}{ccc} 1 & 2 & 1\\ & 1 & 2\\ & & 1 \end{array}\right]$ (different parameters can be chosen in other applications), and (*a*, *b*, *c*) can be obtained using the improved SHA1. Then, *h* = DFEC03870B9D479F7E56AD6A0CECE62724D8F984, $\left\{\begin{array}{c} a=D8F984\\ b=E62724\\ c=6A0CEC \end{array}\right.$. Figures 6(b) and 6(d) are the contrary between the original minutiae and transformed minutiae from the same fingerprint image in Figure 6(a).

### 3.3. The Fingerprint Encryption Algorithm

*n* positive integers which are relatively prime to each other are chosen in the encryption processing. During the decryption processing, CRT is used to obtain the private key from the pair set (*a*_*i*_, *f*(*a*_*i*_)). Assuming that the transformed minutiae of Section 3.2 are (*x*, *y*, *θ*), the combination of *x*, *y*, *θ* in series [*x*∣*y*∣*θ*] is defined as *a*_*i*_, whose length is 24 bits.

#### 3.3.1. The Private Key Combining

Assume that the private key is *K*. Let *m*_1_, *m*_2_,…, *m*_*n*_ be *n* positive integers which are relatively prime to each other. One has *M*_0_ = *m*_1_, *m*_2_,…, *m*_*n*_, *M*_*i*_ = *M*_0_/*m*_*i*_  (1 ≤ *i* ≤ *n*).

Define *k*_*i*_ = *K*mod⁡*m*_*i*_  (*i* ∈ [1, *n*]). (*k*_*i*_, *m*_*i*_) is called a recovering pair. Take *k*_*i*_ as coefficients to obtain the polynomial *f*(*x*) = ∑_*j*=0_^*n*−1^ *k*_*j*_ *x* ^*j*^ = *k*_0_ + *k*_1_ *x* + ⋯+*k*_*n*−1_ *x* ^*n*−1^ and use the pair (*a*_*i*_, *f*(*a*_*i*_)) to form the real minutiae set *C* = {(*a*_*i*_, *f*(*a*_*i*_))}_*i*=1_^*l*^. *l* is the number of real minutiae.

Choose the noise pair (*b*_*i*_, *c*_*i*_) and make sure *b*_*i*_ ≠ *a*_*i*_, *f*(*b*_*i*_) ≠ *c*_*i*_. Then, obtain the fake minutiae set *P* = {(*b*_*i*_, *c*_*i*_)}_*i*=*l*+1_^*t*^. *t* is the sum of fake minutiae and real minutiae. Then, define the vault set as *V* = *C* ∪ *P* = {*X*_*i*_, *Y*_*i*_}_*i*=1_^*t*^.

#### 3.3.2. Private Key Recovering

Compare the fingerprint minutiae after being transformed in Section 3.2 with minutiae in vault *V*. If there are coincident minutiae between these two sets, then these minutiae should be put into the unlocking set *U*. If there are over *n* minutiae in *U*, the legal user can rebuild the polynomial *f*′(*x*) = ∑_*j*=0_^*n*−1^ *k*_*j*_′*x* ^*j*^ = *k*_0_′ + *k*_1_′*x* + ⋯+*k*_*n*−1_′*x* ^*n*−1^ and then use CRT to recover *K* through the recovering pair (*k*_*i*_′, *m*_*i*_).

## 4. Experimental Analyses

This chapter has discussed the efficiency and the security of irreversible transforming and the security of fingerprint encryption algorithm.

### 4.1. The Efficiency Analysis of Improved SHA1

**Figure 7:**
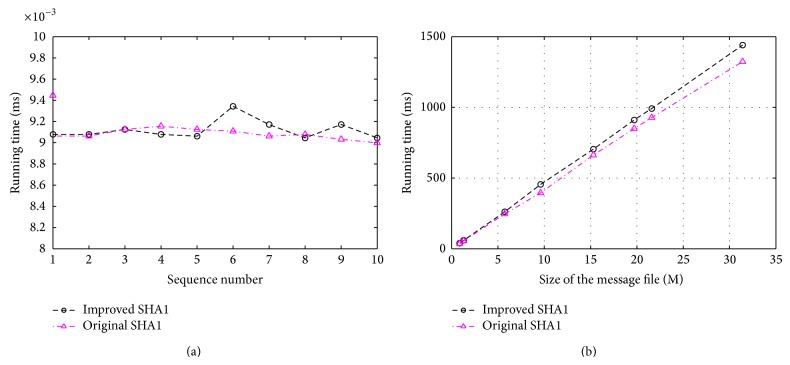
(a) The comparison between improved SHA1 and original SHA1. (b) The efficiency of improved SHA1 and original SHA1.

In the improved SHA1, the complexity has been slightly increased because of the extra circularly left-shift and additional calculation. In a computer with Intel (R) Pentium (R) D CPU 3.0 GHz and RAM 512 M, the hash value is calculated for the same character string “iscbupt” with two different hash functions. The running time of improved SHA1 and original SHA1 has been shown in Figure 7(a).

The running time is different when we use different inputs. According to Figure 7(b), the running time of SHA1 increases linearly when the file size is increased. Comparing to the original SHA1, the running time of improved SHA1 has been increased by nearly 8%.

### 4.2. Security Analysis of Irreversible Transforming

Reference [13] indicates that properties of irreversible transforming are required as follows.

(1) Irreversibility: if the attacker has no idea of the transforming function and the parameters, he cannot recover the original fingerprint vault from the transformed fingerprint vault. The differential in the original SHA1 can be transferred to the highest bit and then be eliminated in some certain steps. Therefore, a collision is found. Relatively, step function (9) in improved SHA1 has two message words *W*[*j*]. According to Table 4, there are two different circularly left-shift calculations which makes it difficult to move the differential to the highest bit and therefore eliminate the differential. Even if the attacker can eliminate 1-bit differential, it will create a 2-bit new differential. If the differential has not been eliminated in time, the formula containing *W*[*j*] will become more complex and make the attacker even harder to find a collision (Table 3).

In this paper, Blank_1_ = 32,30,10,8, 26,6, Blank_2_ = 17,15,27,25,11,23.

In addition, since the irreversible function contains module calculation, the data has been transformed from the domain (0 ~ 2^24^) to a smaller domain (0 ~ 256). This reflection from a domain to a smaller domain can increase the uncertainty. It seems that the irreversible function proposed by this paper has great irreversibility. The security proof has been shown as follows.

According to Section 2.2, the most important property of a hash function is anti-strong collision. In other words, if the hash function possesses the property of anti-strong collision, it also possesses the property of irreversibility.

Let $\chi =r\sqrt{m}$, *s* = (*a*, *b*, *c*)^*T*^, and $p\geq 4\sqrt{m}k^{1.5}\chi$; *M*′ ∈ *Z*_*p*_^*k*×*m*^ is a random matrix with the size of *k* × *m*, and *M* = *λM*′ ∈ *Z*_*q*_^*k*×*m*^(*λ* = *q*/*p*). If there is an algorithm which can find two different vectors *v*_1_, *v*_2_ ∈ {0,1,…,*q* − 1}^*m*^, which make *Mv*_1_ + *s* = *Mv*_2_ + *s* (mod⁡ *q*), then there must be an algorithm which can solve all cases in $GAPSVP_{14\pi \sqrt{k}\chi }$.

These two different vectors *v*_1_, *v*_2_ ∈ {0,1,…,*q* − 1}^*m*^ make *Mv*_1_ + *s* = *Mv*_2_ + *s* (mod⁡*q*). Then, *M*(*v*_1_ − *v*_2_) = 0 (mod⁡*q*). The definition of matrix *M*′ ∈ *Z*_*p*_^*k*×*m*^ can be described as

(12) λM′v1−v2=0mod⁡λp⟹∃t∈Zk: λM′v1−v2=λtp⟹M′v1−v2=0mod⁡p.

Let $z=v_{1}-v_{2},\left\|z\right\|\leq \chi ,\chi =r\sqrt{m}$. Since *M*′ ∈ *Z*_*p*_^*k*×*m*^ is random and the coordinate of *z* is between −(*q* − 1), +(*q* − 1), *z* is a solution to *SIS*_*p*,*m*,*χ*_. According to Section 2.4, this solution can solve all cases in $GAPSVP_{14\pi \sqrt{k}\chi }$. However, there is no such algorithm at present and the irreversible function proposed by this paper is antistrong collision.

(2) Local smoothness: in order to decrease the FRR, the transformation should guarantee that a small change in the original fingerprint image will also cause a small change in the transformed fingerprint image. Fingerprint recognition is a fuzzy technique. Due to the influence from moving, sweat, different finger pressure, orientation difference and noise, and so forth, there are different for the extracting results in every two different experiments from the same fingerprint. However, as cryptology is an accurate technique, a tiny error can lead to the failure of decryption. How to combine the accuracy of cryptology with the fuzzy property of fingerprint recognition becomes more and more important. A relatively small threshold is required. The two minutiae can be matched if the difference between the registered minutiae and the authentication minutiae is smaller than this threshold. This matching method is called the tolerance box matching algorithm.

**Figure 8:**
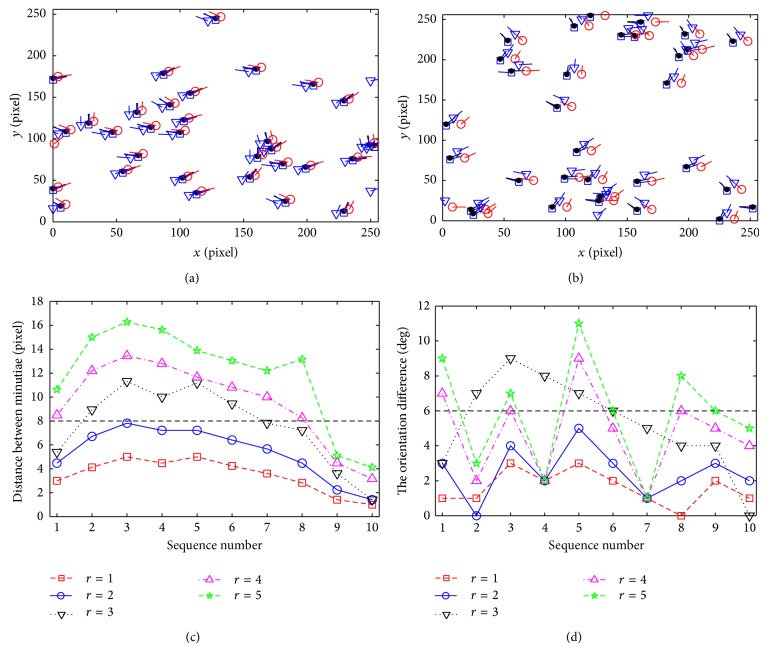
The comparison of four fingerprint templates in three different authentication experiments using different *r*. (a) Three transformed templates with the same fingerprint when *r* = 2. (b) Three transformed templates with the same fingerprint when *r* = 4. (c) The distance between transformed and original templates. (d) The orientation difference between transformed and original templates.

The value of *M* is fatal to the local smoothness property. Let the maximum element of *M* be max⁡ (*m*_*i*__*j*_) = *r*. Figures 8(a) and 8(b) show the comparison among the four fingerprint templates from three different authentication experiments (there will be difference among the authentication processing) and the registered fingerprint template when their *r* have different values. Figures 8(b) and 8(d) show the distance between minutiae and the orientation difference from transformed and original templates. According to Figure 8, if *r* = 2, the three fingerprint templates transformed from the same fingerprint in three different experiments are almost coincident with each other. The distance between minutiae of these three templates and the registered template is less than 8 pixels, and the orientation difference is less than 5°. If *r* = 4, the distance between minutiae of these three templates and the registered template is less than 14 pixels, and the orientation difference is less than 9°. According to Table 5, with the increasing of *r*, the distance between minutiae and the orientation difference will be increased linearly. Let *r* ≤ 2; then, the average distance of minutiae between two templates is no more than 5.4 pixels, which is less than the box matcher threshold (8 pixels). This can ensure that the legal user can pass the authentication processing using the tolerance box matching algorithm.

**Figure 9:**
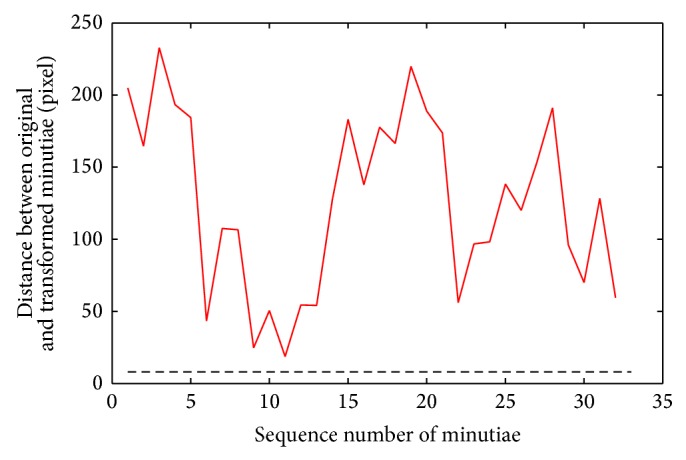
Distance between transformed and original minutiae.

**Figure 10:**
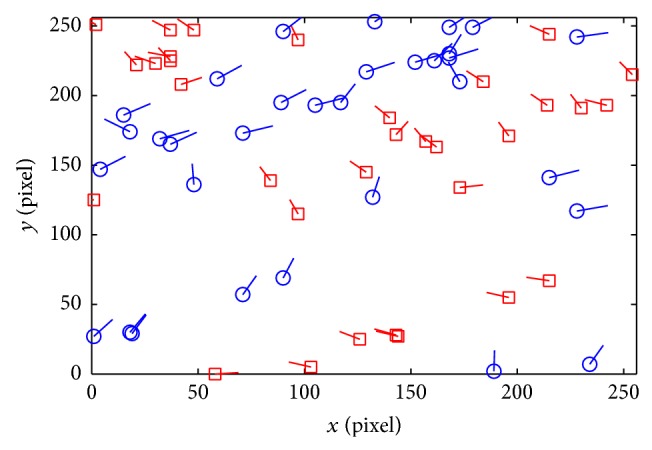
The comparison between 2 transformed templates with the same *M* and different *R*.

**Figure 11:**
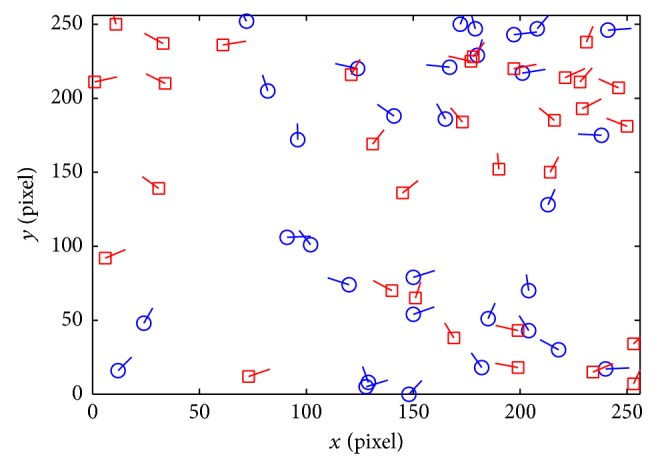
The comparison between 2 transformed templates with different *M* and different *R*.

(3) Transformation: the transformed minutiae must be outside the matcher tolerance box and cannot be matched with the original minutiae. According to Figures 6(d) and 9, the transformed minutiae are totally different from the original ones. The distance is far farther than 8 pixels. And it is hard to find the relativity of these two templates. Therefore, the irreversible function proposed by this paper can satisfy this principle.

(4) Distinctiveness: it cannot be matched to each other for the different transformed templates from the same original fingerprint template applied in different systems. Otherwise, the system will suffer from multiple templates attack. According to Figures 10 and 11, if the same matrix *M* and yet different random number *R* have been used in the transformation, the coordinate of fingerprint minutiae transformed fingerprint templates has great distinctiveness characteristic. However, the orientation still has certain relativity. When different matrix *M* and different random number *R* have been used, not only the coordinate but also the orientation information will have great distinctiveness property.

To sum up, even if the attackers have obtained the right “minutiae” from the transformed fingerprint template, they cannot use the transformed minutiae to recover the original fingerprint template since the system has the distinctiveness and irreversibility properties. After transformation, the fingerprint minutiae are no longer related to the original minutiae, and the system can resist the multiple templates attack. In addition, the fingerprint templates are cancelable when the system is under attack. A new template can be rebuilt through creating new parameters (random number *R* and matrix *M*).

### 4.3. Security Analysis of Encryption Algorithm

The international standard fingerprint database FVC2002 has been chosen to verify the efficiency of the scheme proposed in this paper. The fingerprint images have been put in the same orientation before the experiment in order to decrease the influence from the image moving and other noises. This section mainly focuses on discussing the performance of the transformed function.

Choose the parameter *n* = 20, *l* = 8. The maximum power *n* in polynomial *f*(*x*) = ∑_*j*=0_^*n*−1^ *k*_*j*_ *x* ^*j*^ = *k*_0_ + *k*_1_ *x* + ⋯+*k*_*n*−1_ *x* ^*n*−1^ is decided by the length of the private key *K* if the length of *K* is fixed and the matching number of minutiae is determined too. Users have to use the recovering pair to obtain *K*. An illegal user does not know *m*_*i*_ (*i* = 1,2,…, *n*). In order to obtain *K*, he has to use the exhaustive method. On the other hand, it is impossible to find the real minutiae in the fingerprint vault through the exhaustive method. The number of noise points is ten times than the real minutiae (200 : 20) in this experiment. The probability that the private key can be recovered from the exhaustive method is *C*_20_^9^/*C*_220_^9^ = 7.05 × 10^−9^%. In order to obtain the private *K*, the user has to unlock the fingerprint vault through providing the legal fingerprint.

The comparison between [6] and this paper is listed in Table 6. The database of both [6] and this paper is the fingerprints in FVC2002 DB2.

According to Table 6, this paper costs less time during verifying process. Its security is based on the well-known $GAPSVP_{14\pi \sqrt{k}\chi }$ problem. At present, there is no effective algorithm to solve this problem. The GAR (genuine accept rate) of this paper is close to [6], and the FAR is much better than [6]. With the increasing of *n*, it becomes harder to get successful authentication. In this case, both GAR and FAR will decrease simultaneously.

## 5. Conclusions

In this paper, an irreversible function has been proposed to protect the original fingerprint template, and the CRT is used for combining the private key with the transformed fingerprint vault. Even if the system is under attack, the irreversible function can also guarantee the security of the original fingerprint after the transformed fingerprint vault was filched. The security analysis shows that the fingerprint encryption system proposed by this paper has better efficiency and security, and the complexity is only slightly increased.

## Figures and Tables

**Table 1:** The contrast between the traditional algorithm and this paper (in 20 images).

	Minutiae detected	Fake minutiae	Lost minutiae	FAR	FRR
Based on thinned fingerprint image	496	66	21	14.6%	4.7%
After removing the fake minutiae	411	4	44	0.9%	9.8%

**Table 2:** An example of local collision.

Step	*A*	*B*	*C*	*D*	*E*	*W*[*j*]
*j*						1
*j* + 1	1					6
*j* + 2		1				1
*j* + 3			31			31
*j* + 4				31		31
*j* + 5					31	31
*j* + 6						

**Table 3:** 

*h* (160 bits)												

160	⋯	73	72	⋯	49	48	⋯	25	24	⋯	2	1

			*c* (24 bits)			*b* (24 bits)			*a* (24 bits)			

**Table 4:** Local collision against improved SHA1.

Step	*A *	*B *	*C *	*D *	*E *	*W*[*j*]
*j*						28
*j* + 1	1,13					1,13
*j* + 2	18,30	1,13				18,30,28,8
*j* + 3	3,15,13,25	18,30	31,11			3,15,26,6
*j* + 4	20,32,11,23	3,15,13,25	16,28	31,11		Blank_1_
*j* + 5	Blank_2_	20,32,11,23	1,13,11,23	16,28	31,11	

**Table 5:** The minutiae difference among many experiments after being transformed.

	*r* = 1	*r* = 2	*r* = 3	*r* = 4	*r* = 5
The average distance between minutiae (pixel)	3.5	5.4	7.6	9.5	11.9
The average orientation difference between minutiae (degree)	1.6	2.5	5.3	4.7	5.8

**Table 6:** The comparison between [6] and this paper.

	Time of verification	The security hypothesis	*n* = 7		*n* = 9		*n* = 11	
			GAR	FAR	GAR	FAR	GAR	FAR
[6]	42 ms	SHA256	96.50	0.30	93.57	0.10	90.05	0
This paper	20 ms	${GAPSVP}_{14\pi \sqrt{k}\chi }$	95.88	0.14	93.35	0.02	89.61	0
